# Chat-based interaction in virtual classes: EFL students’ perceptions, motivation, and well-being

**DOI:** 10.3389/fpsyg.2026.1807648

**Published:** 2026-08-25

**Authors:** Nada Fahad Bin Dahmash

**Affiliations:** College of Applied Studies, King Saud University, Riyadh, Saudi Arabia

**Keywords:** chat-based interaction, computer-mediated communication (CMC), EFL learners, motivation, perceptions, synchronous virtual learning, well-being

## Abstract

**Background:**

Despite the growing body of research concerning online learning technologies in language education, limited attention has been directed toward realizing the affordances of chat-based interaction in a synchronous virtual classroom. This study explores the perceptions of female EFL postgraduate students regarding the use of chat-based interaction in synchronous virtual classrooms, specifically in reference to their motivation and well-being after attending a 15-weeks intensive English course delivered online via Blackboard.

**Methods:**

A cross-sectional survey design with a quantitative research methodology was used to collect data from (*N* = 128) female Saudi postgraduate students enrolled on an online English course for 15 weeks. It drew on correlational methodology to identify any relationships with the student’s demographic information and their perceptions, motivation, and well-being. The data were analyzed using descriptive statistics, reliability tests, Pearson correlations, a linear regression model, and an independent samples *t*-test, and Welch’s one-way ANOVA.

**Results:**

The findings demonstrate a consistent tendency for postgraduate EFL learners to report positive experiences with chat-based interaction in the synchronous virtual classroom. Their perceptions, motivation and well-being were strongly and significantly interrelated, with motivation exhibiting the highest relationship to well-being (*r* = 0.867, *p* < 0.001); (*B* = 0.734, *β* = 0.704, *p* < 0.001). The EFL students’ experience using Blackboard virtual classes was associated with a statistically significant difference in their well-being (*p* = 0.016, *p* < 0.05); there was no significant difference in any construct by academic major or English-learning experience; age showed a significant omnibus effect on motivation (*p* = 0.020), attributable to a single pairwise contrast between the youngest and oldest age groups, but no significant effect on perceptions or well-being.

**Conclusion:**

The results revealed chat-based interaction is an accommodative, low-anxiety communication method that is associated with engagement and well-being among learners in online EFL settings. It adds to the prevailing literature by promoting knowledge of chat-based interaction as a psychologically and pedagogically significant mode of communication. It further emphasizes the importance of instructors including chat-based activities to create supportive, engaging, and emotionally safe learning environments in virtual classrooms. Given evidence of substantial conceptual and empirical overlap among the three constructs (see Instrument Validity), these findings should be interpreted as exploratory and correlational rather than confirmatory or causal.

## Introduction

The burgeoning field of online education has transformed the landscape of higher education worldwide. Virtual learning has moved beyond its role as a crisis response method, to become a mainstream instructional modality. Following the COVID-19 pandemic, several universities and educational institutions continued with synchronous online teaching as they had come to value its flexibility, accessibility, and efficiency (Amekhlafy and Alqahtani, 2022). This shift has significantly altered patterns of classroom interaction, learner engagement, and emotional experiences in the language learning environment. Although online learning is both interactive and convenient, barriers to its success can include communication anxiety, reduced immediacy, and low rates of interaction. These factors are especially applicable in the second language learning environment, where communication is the main component of learning (Heift and Chapelle, 2012; Lightbown and Spada, 2013).

Interactions in the English as a Foreign Language (EFL) classroom are crucial to the development of a foundation for learners’ progress. Interactionists and sociocultural perspectives recognize the fact that the learners’ knowledge is constructed in response to exposure, requiring negotiation, feedback, and socially mediated interaction (Long, 1996; Swain, 2005; Vygotsky, 1978). Interactive activities will enable learners to practice various forms of language, improve their knowledge of their mistakes, and be guided by their peers and instructors. Traditionally, interactions in virtual classrooms may be disrupted by learners’ technological weaknesses, low social presence, and elevated levels of anxiety, particularly when oral participation is required. It has been empirically proven that synchronous text-based interaction can reduce anxiety and enhance willingness to communicate when compared to voice interaction (Knežević et al., 2025; Namaziandost et al., 2021). These findings confirm the importance of researching the use of alternative communicative media in synchronous classes.

Blackboard has emerged as the principal system of synchronous delivery at the majority of universities in Saudi Arabia. The system provides different communication media, such as audio, video, and text-based media, through chat. Participation by chat affords a simple format, in which EFL learners can provide their own answers, and quiet learners can be encouraged to participate, avoiding the stress of answering in front of a group. Saudi-based research claims that students are likely to believe Blackboard can be useful as a tool for ensuring attachment, feedback, and language development (Al-Fraihat et al., 2020; Al-Qahtani, 2019; Bervell and Arkorful, 2020). To date, however, most studies have examined Blackboard at the system level, with little exploration of how the mode of interaction affects the learning experience.

The interactions of learners online are also determined by motivation. This has an impact on hard work, perseverance, and long-term involvement in learning processes (Ortega, 2013; VanPatten and Benati, 2015). Self-Determination Theory (SDT) identifies autonomy, competence, and relatedness as psychological needs that influence motivation (Ryan and Deci, 2000), and studies on language learning further emphasize the significance of contextual factors (Dörnyei, 2005). Depending on the instructional design, social support, and perceived quality of the interaction, virtual classrooms can be relatively more or less motivated than offline settings. Recent studies confirm the benefits of the online supportive environment in terms of the degree of engagement and persistence in language learners (Jiang et al., 2024; Xu and Rahim, 2025).

The well-being of learners, together with their motivation, has emerged as one of the most important constructs in educational research, especially in higher education. Well-being informs the extent of positive emotions, positive feelings, positive relationships and personal development (Hako and Mbongo, 2024). The scientific literature in the domains of applied linguistics is focused on positive psychology, with emotional development and prosperity among students valued as more important than minimizing anxiety (MacIntyre et al., 2019; Seligman, 2011; Seligman and Csikszentmihalyi, 2000). Flexibility and autonomy can be used to sustain the well-being of students in distance learning, with the absence of physical interaction and interpersonal bonding posing a threat. The empirical research confirms that independence, resistance, and interaction can be significant predictors of well-being in EFL online environments (Wang and Wu, 2025; Zhou and Liu, 2025).

Despite the increasing body of research on the topic of online learning technologies, the majority of research has evaluated either the efficiency of the platform, user satisfaction, or course design. Relatively few studies have researched chat-based interaction as a communication mode, per se, in synchronous classrooms. Indeed, literature exploring how this mode relates to both motivation and well-being is even rarer (Amekhlafy and Alqahtani, 2022; Knežević et al., 2025). Consequently, there has been a lack of thorough theorization of the psychological and pedagogical usefulness of chat-based interaction online.

To be more precise, this lack of connection may be observed among postgraduate EFL students in Saudi Arabia. Although the existing literature assumes the implementation of Blackboard as a natural process of change within institutions (Al-Fraihat et al., 2020; Al-Nofaie, 2020; Al-Qahtani, 2019), the direct experience of learners who interact with other participants in synchronous classes through text chat has not been properly examined. Moreover, Saudi postgraduate women students have not been sufficiently represented in the literature, despite the fact that their cultural and educational context uniquely influences interaction patterns.

Thus, this study seeks to identify the perception of female EFL postgraduate students engaged in chat-based interactions in synchronous virtual classrooms, examining associated levels of motivation and well-being. It hypothesizes whether positive perceptions of chat-based interactions correlate with motivation and lower psychological stress, and any association with specific learner features. Further engagement in chat-based interactions is assumed to indicate additional motivation and improved well-being.

Addressing this gap will be useful, since the perceptions of chat-based interaction, motivation, and well-being will be combined in a single analysis. Practically, it teaches teachers, institutions, and policymakers interested in improving levels of engagement and emotional support among learners in synchronous online learning environments (Zhou and Liu, 2025). The study findings would assist with justifying evidence-based decisions in the pedagogic application of chat-based interactions when teaching English as foreign language (EFL).

## Literature review

### EFL education in virtual setting

The increasing trend toward the adoption of online educational tools has fundamentally changed the method by which language is being taught, especially since the COVID-19 pandemic, when the majority of institutions were compelled to implement remote instruction. What was initially a crisis response is now a formalized mode of teaching used throughout higher education. Researchers now distinguish between emergency distance learning during the pandemic and planned online teaching, highlighting that the former was aimed at short-term accessibility, and did not prioritize teaching quality (Adedoyin and Soykan, 2020). Virtual classrooms present an opportunity for flexibility and control by the learner in EFL context; however, they also present a challenge in terms of instruction design, interactions with learners and practical implementation (Means et al., 2014).

It has been suggested that, while online settings offer continuity in terms of language learning, there may be limitations on spontaneous interactions in situations where learners can use the language more naturally (Heift and Chapelle, 2012). The aim is meaningful interaction; as the interactionist perspective argues, interaction is crucial to second language acquisition as learners develop through language exposure, negotiation with others and linguistic feedback (Long, 1996). In cases where there is reduced use of online space, communicative competence is likely to be compromised. Lightbown and Spada (2013) emphasize that development processes rely on continuous input, significant output, and corrective feedback, and may be disrupted in online courses when a learner and a teacher are not interacting.

It has also been proven that EFL students can experience emotional problems in the virtual environment, including anxiety, inability to concentrate, and isolation. Students may be more willing to participate in communication processes in textual format than the oral format due to fears of receiving a negative evaluation, potential technical problems, and perceived social inadequacy (Namaziandost et al., 2021). Sociocultural theory also postulates that interaction causes mediation and social influence when individuals are learning through interactions with others (Vygotsky, 1978). Consequently, the Internet-based EFL classroom should be equipped with a pedagogical strategy that promotes communication and facilitates emotional involvement among learners.

### Blackboard in higher education

As mentioned previously, Blackboard is one of the most widespread learning management systems (LMS) used in Saudi higher education to teach through synchronous online delivery. It provides teachers with the opportunity to use video, audio, and written messages to conduct live classes. According to regional and international research, LMS platforms such as Blackboard, are convenient and efficient tools to facilitate continued learning (Al-Fraihat et al., 2020; Hussain et al., 2018). Faculty members typically report positive experiences when using LMS platforms to assist with integrating online and offline instruction (Bervell and Arkorful, 2020).

The platform supports instruction through the ability to record lessons, breakout rooms, screen sharing, and chat. When used appropriately, these educational aids accommodate distinct types of learning and make interactions easier. Bervell and Arkorful (2020) found users were happy with the Blackboard platform, because it provides flexibility and control over educational content, as well as accessibility for students. However, there are still concerns related to technological complexity and cognitive overload. According to research by Al-Nofaie (2020), students experienced problems with the utilization of tools offered by the platform, or with focused attention in synchronous classes. Similarly, Al-Qahtani (2019) observed that the efficiency of platforms depends on their pedagogical design, but not necessarily on the functionality of the system.

Pedagogical research is also considering platform efficacy in the context of theoretical engagement models, such as the Community of Inquiry model, which views social presence, cognitive presence, and teaching presence as the basic features of an online learning environment (Anderson, 2008). Martin and Bolliger (2018) further assert that interactions between the design and engagement of learners in the virtual classroom is not only based on access to digital tools but also on interactive design. Thus, the educational merit of Blackboard should be viewed from the perspective of its ability to support interaction, rather than concentrating on availability.

### Chat-based interaction in online learning

Oral interactions can affect the effectiveness of text-based communication in real-time online classes. Chat options facilitate communication with the learner through written communication during live instruction, without disruption. This mode assists EFL learners to be more mindful when processing language and reduces cognitive burden in cases of spontaneous speech.

Research in the field of computer-mediated communication has always confirmed written communication assists with negotiating meaning and output among learners (Smith, 2004; Warschauer, 1997). Seen alongside the output hypothesis, it is apparent that language productions make it possible to highlight any linguistic weaknesses and build more profound processing (Swain, 2005). Active engagement reportedly also leads to reduced anxiety and an increased willingness for students to communicate among themselves using text (Knežević et al., 2025; Namaziandost et al., 2021). The results of such findings lend credence to the notion that chat-based interaction relieves the pressures associated with pronunciation and urgency in oral communication.

When engagement is facilitated through chat-based interaction, silent learners can feel relaxed about participating without the feeling of being interrupted. Amekhlafy and Alqahtani (2022) have found that the learners also engaged in academic clarification of chat functions which supported socialization and contributed to the formation of a community. Chat is an instructional tool rather than a technology, with the chat skills in the Computer Assisted Language learning (CALL) approach enabling a higher level of interaction than traditional oral interactions (Blake, 2011).

It is important to distinguish chat-based interaction, as a specific communicative mode, from the broader virtual-classroom or Blackboard experience discussed in the preceding subsection. Much of the literature reviewed above on Blackboard and LMS platforms addresses system-level satisfaction, usability, and design (e.g., Al-Fraihat et al., 2020; Bervell and Arkorful, 2020), without isolating the specific contribution of text-based chat as a communicative channel within that broader experience. Chat-based interaction is conceptually distinct in that it is a specific mode of synchronous computer-mediated communication (CMC) with properties; reduced real-time production pressure, increased planning time, and text-based rather than oral output, that are not shared by other virtual-classroom features such as video, breakout rooms, or screen sharing. These properties are theorized to lower learner anxiety and support social presence specifically through the written, asynchronous-feeling nature of text exchange (Reinders and Wattana, 2015; Warschauer, 1997), independent of how learners evaluate the platform or course as a whole. Because prior research has largely evaluated Blackboard or online learning as an undifferentiated whole, the present study treats chat-based interaction as an analytically separate construct from general course or platform satisfaction; this distinction motivates the present focus on chat specifically, though it also means that learners’ responses to our questionnaire may still be partly colored by their broader course experience, a possibility the researcher returns to in the Limitations.

### Motivation of language learning

The persistence and success of learners is highly dependent on motivation. According to Ortega (2013), the factor that keeps the process of learning going is intrinsic motivation, also known as goal motivation by VanPatten and Benati (2015). Motivated students in language classes tend to participate and engage actively in assigned tasks and show interest in the content and study harder (Lightbown and Spada, 2013). Online learning is particularly vulnerable to drops in motivation, as the need for autonomy is paramount when supervision by teachers is minimal.

Self-Determination Theory (SDT) not only differentiates between intrinsic and extrinsic motivation, it also emphasizes three key needs: autonomy, competence, and relatedness as central to governing engagement (Ryan and Deci, 2000). Dörnyei (2005) also contributes to the fact that motivation is socially constructed and is influenced by learners’ background. Environments that are based on interaction encourage intrinsic motivation by facilitating the agency and interest of the learner.

The academic evidence is supported by the fact that positive virtual worlds are motivating. Jiang et al. (2024) discovered that peer interaction and an instructor’s presence are significantly affected by engagement. Xu and Rahim (2025) determined that digital social support increased learners’ persistence. Their involvement in chat also enhances self-efficacy by providing a meaningful contribution, which improves perceived competence.

### Psychological well-being in language learning

Sustainable learning involves the psychological well-being of learners. The notion of well-being as set out by Zhou and Liu (2025) relates to enjoyment, connectedness, meaning, and competence. Enjoyment refers to learners’ engagement in the language classroom and their positive emotions, connectedness refers to positive social ties, meaning refers to their inner value orientation toward the language learning process and competence refers to their ability and confidence in using the language. This conforms to models of positive psychology, which appeal to flourishing learners (Seligman, 2011). Positive emotional conditions promote activity, energy and engagement.

MacIntyre et al. (2019) suspects that learning should be designed not to reduce anxiety but rather to enable psychological growth. The empirical studies available demonstrate that autonomy, resilience and social support are good predictors of well-being in online learning (Wang and Wu, 2025). The fear of evaluation related to chat-based interactions can be reduced, to provide emotional safety and encourage participation.

### Empirical studies

The empirical research on this topic in Saudi Arabia has addressed the analysis of Blackboard as an LMS rather than the critical examination of the modalities of communication per se. Al-Fraihat et al. (2020) observed that the motivation of the learners was greater when Blackboard was used as an online platform, but the authors did not differentiate between aspects of the instructions on the Blackboard in their analysis, and the role of the chat-based interaction in this scenario remained undiscovered. Similarly, Hussain et al. (2018) indicated that learning analytics for LMS systems can predict and optimize student interaction and learning. As Gaffas (2023) observed, the virtual online classroom proved better in terms of interactions but did not explore how certain modes of communication might contribute to engagement.

Chat-based interaction is an aspect that has not yet been directly studied in Saudi research. Alqasham (2022) investigated the process of collaborative writing through the use of Blackboard chat box and reported positive emotions among students; but addressed the linguistic aspects of writing more than the psychological ones. Meanwhile, Amekhlafy and Alqahtani (2022) provided the results of a study that found chat communication helped learners to engage in academic activity and communicate with their peers, which suggests the non-conventional pedagogical significance of text communication.

This is also reflected in international research in this area. The affective advantage of written interaction in virtual classes was demonstrated by the finding that text-mediated interaction was significantly less anxiety-inducing than voice contact (Reinders and Wattana, 2015). You et al. (2024) associated online platforms with an increase in speaking, but did not specifically identify chat-based interaction as an instructional factor. Collectively, the findings suggest that, although interactions through chat benefited learners, it is a poorly articulated and poorly tested instructional mode.

A note on construct framing is warranted here. Perceptions, motivation, and well-being are treated as three conceptually distinct constructs in this study because they are theorized separately in the literature reviewed above: perceptions reflect learners’ evaluative beliefs about chat-based interaction as a communicative tool, motivation reflects the psychological drive to engage with it grounded in Self-Determination Theory (Ryan and Deci, 2000), and well-being reflects learners’ affective and emotional experience of using it. These are conceptually separable processes even though, as this study’s own factor-analytic findings later indicate (see Instrument Validity subsection), they are empirically highly correlated in practice. The researcher retains the three-construct framing for continuity with this theoretical distinction and with the wider literature that treats these as separate outcomes, while acknowledging and addressing their empirical overlap directly in the Results, Discussion, and Limitations.

### Purpose of the present study

Even though online education is gaining in popularity, there is insufficient evidence in the literature regarding how learners interact in virtual classrooms. The focus has previously been, not on the value of specific interaction modes, but rather on the usability of the system or users’ overall satisfaction. As a result, the conceptual development of chat-based interaction is still lagging behind in synchronous EFL learning research. Moreover, motivation and well-being research has largely been conducted independently of these two constructs. The literature combining the two variables in the same study is very meager constraining theoretical explanations as to the main role of motivation and well-being processes in the context of virtual language learning. Empirically, Saudi postgraduate learners, particularly female learners, are underrepresented in empirical studies. The available literature is primarily about undergraduates or general student groups, and postgraduate experiences are not covered.

The purpose of the present study is to bridge three gaps; a conceptual gap (a theoretical framework evaluating chat efficacy has not been formulated), a methodological gap (minimal quantitative research on relational studies is available), and a contextual gap (there are no female Saudi postgraduate opinions available). To fulfill this purpose, four research questions were formulated:

1. What are EFL postgraduate learners’ perceptions, motivation levels, and well-being regarding chat-based interaction in synchronous virtual classrooms?
2. How do EFL postgraduate learners’ perceptions of chat-based interaction in synchronous virtual classroom relate to their motivation levels and well-being?
3. To what extent do EFL postgraduate learners’ perceptions and motivation to engage in chat-based interaction predict their well-being in virtual learning environments?
4. Do students’ perceptions, motivations, and well-being differ in relation to demographic variables?

## Materials and methods

### Research design

The research design implemented in this study was a quantitative, cross-sectional, correlational, and exploratory survey design. The tool used was a questionnaire, as this is a valuable instrument with which to measure attitude and psychological variables for a large population in the field of applied linguistics (Dörnyei and Taguchi, 2010; Heift and Chapelle, 2012). The use of correlational methodology in this study was appropriate, as there was no intention to control or manipulate variables, as the focus was on measuring and identifying the strength and direction of the relationships between learner traits that occur naturally (Giovannone and Toscano, 2024; Rahimi and Sevilla-Pavón, 2024).

### Participants

The participants were 128 postgraduate students who had studied on a compulsory intensive English skills course delivered via Blackboard. All the participants were native Arabic speakers and Saudi women studying at a university in Saudi Arabia. The students had direct experience engaging in chat-based interaction during synchronous virtual classes as they had participated in the classes throughout the 15-week semester.

The participants were recruited applying purposeful sampling (Palys, 2008). They had each studied a compulsory intensive English course on one of two different high diploma programs: Human Resources Management (28.9%), and Governance and Internal Audit Assistant Specialist (71.1%) at a Saudi university. They were of different ages, with 34.4% aged 23–27, 33.6% aged 28–32, 19.5% aged 33–37, and 12.5% aged 38 and above. Moreover, the participants had different English-learning experience, and varied prior experience of virtual classes, which allowed to draw significant comparisons between the demographic groups (see Table 1). The use of purposive sampling from a single institution limits the generalizability of these findings to other populations and contexts.

**Table 1:** Participants’ demographic information.

Variable	Items	*n*	%
Age	23–27	44	34.4%
	28–32	43	33.6%
	33–37	25	19.5%
	38+	16	12.5%
Experience with using virtual classes on Blackboard	Less than 6 months	55	43%
	More than 6 months and less than 1 year	13	10.2%
	1 year +	60	46.9%
Experience learning English	Less than 3 years	54	42.2%
	3–8 years	44	34.4%
	8 years +	30	23.4%
Current major	Higher Diploma in Human Resource Management	37	28.9%
	Higher Diploma in Governance and Internal Audit Assistant Specialist	91	71.1%

### Instrument

The questionnaire statements were devised by the researcher based on a literature review of online EFL interactions and learner psychology studies (Alqasham, 2022; Amekhlafy and Alqahtani, 2022; Wang and Wu, 2025). The researcher also drew on her own previous experience teaching English synchronously in virtual classrooms to bridge gaps in the literature.

The study used a structured questionnaire format with closed-ended five-point Likert-scale items, where 1 indicates (“Strongly Disagree”), and 5 indicates (“Strongly Agree”). The questionnaire was comprised of four main parts: the first part collected the participants’ demographic information, including their ages, experience in virtual classes, experience learning English and their academic major. The second part (ten statements) collected details of their perceptions of chat-based interaction. The third part (ten statements) considered their motivation to engage in chat-based interactions, while the fourth part (ten statements) considered their well-being when engaged in chat-based interactions.

The questionnaire was originally prepared in English and reviewed by three applied linguistics experts to ensure content validity, clarity and compliance of the concepts with the constructs that the questionnaire aimed to measure in line with common practice (Taherdoost, 2016). The questionnaire was translated into Arabic to make it easy for the participants to understand and later translated back into English to validate the quality of the questionnaire and ensure accuracy, which is advised during multi-lingual survey research (Brislin, 1970). Then, the Arabic version of the questionnaire was piloted with 10 students from the target population to ensure the instrument’s content validity; in particular that the items were clear, easy to understand and appropriate. The pilot sample (*n* = 10) is relatively small for establishing content validity and should be considered a limitation of the instrument development process. In response to feedback, minor phrases were rephrased to make them easier to read.

The internal consistency reliability of the piloted instrument was measured using the Cronbach’s Alpha coefficient determined by statistical software (SPSS) (Cronbach, 1951; Field, 2024). The questionnaire items from 1 to 30 were found to have high reliability (*α* > 0.90), above the recommended 0.70 for psychological tools (DeVellis and Thorpe, 2022; Nunnally and Bernstein, 1994).

The final questionnaire was presented to the Institutional Review Board at the participants’ university, to obtain permission before distributing the questionnaire to the target population. Approval for conducting research involving human participants was granted by the committee at the participants’ university (reference number: KSU-HE-25–1,427).

### Procedures

The participants engaged in chat-based interactions on synchronous virtual classes on Blackboard from week 1 to week 15 when attending an intensive English course. The teacher invited the participants to engage in speaking skills discussions, answer listening, writing and reading skills tasks, answer grammar tasks, and post clarification questions using either their voices or text in the chat box.

The researcher invited all the students (*n* = 198), who had attended the online sessions of intensive English course that lasted 15 weeks, to fill in an online questionnaire; of these (*n* = 128) completed the questionnaire (a response rate of 64.6%). The questionnaire link was administered on Google Forms and distributed via the students’ WhatsApp groups in December 2025, and the researcher reminded the participants to fill in the questionnaire every day for ten successive days. On the seventh day only one participant filled in the questionnaire, and none filled in the questionnaire on the 8th, 9th or 10th day. The questionnaire included information about the research to ensure informed consent and the participants were informed that their participation was voluntarily, data would be anonymized and used for research purposes only and that no risks were predicted as a result of completing the questionnaire. The participants had to select “I agree,” if they consented to participate in the study, this would then take them to the next page where the questionnaire content was displayed. The researcher was also the course instructor; however, the 15-week course had concluded before the questionnaire was administered, and completion was entirely voluntary. Responses were anonymous and the researcher could not identify individual students from their responses, in line with the ethics committee’s requirement that participant identities remain anonymized. Students were given complete freedom to respond and were reminded, but not required, to do so. The researcher nonetheless acknowledges that the researcher–instructor relationship, recruitment through students’ own class WhatsApp groups, and repeated daily reminders raise a plausible risk of demand characteristics and social-desirability bias; this risk is discussed further through the sensitivity analysis reported in the Results section, which excludes straight-line responders; however, that analysis addresses inattentive responding and does not by itself rule out social-desirability effects among attentive respondents.

### Data analysis

**Figure 1:**
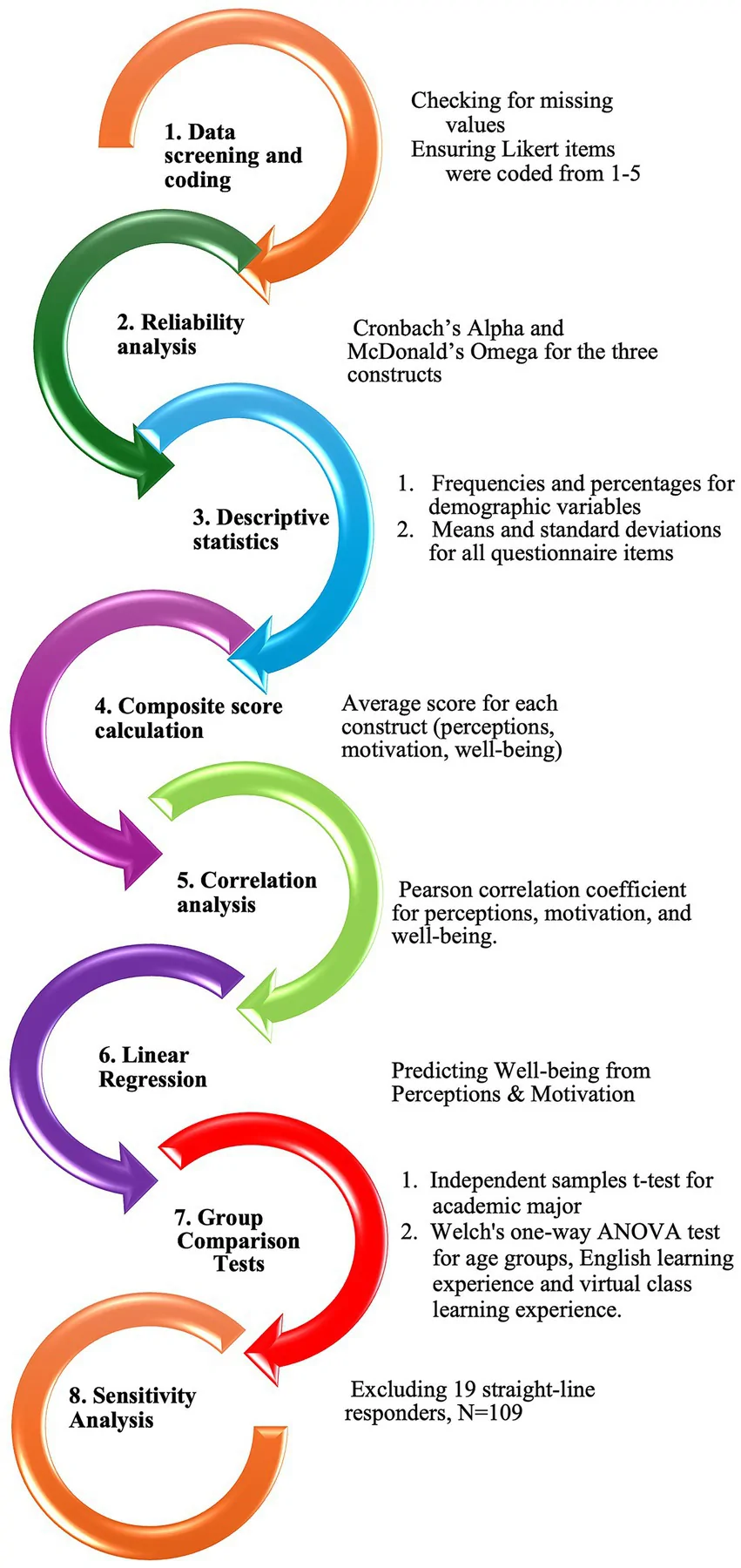
Data analysis procedure.

The statistical software Jamovi 2.7 (The Jamovi Project, 2025) was employed to analyze the data and the procedure illustrated in Figure 1 was applied. These tests are commonly used in applied linguistics and educational psychology research to examine attitudinal and psychological variables (Larson-Hall, 2015; Rietveld and Hout, 2005). Descriptive statistics detailing means and standard deviations (SD) values were used to answer question (1) where the level of approval of the participants’ perceptions, motivation and well-being were categorized in degrees as follows: very low degree 1.00–1.80; low degree 1.81–2.60; medium degree 2.61–3.40; high degree 3.41–4.20; and very high degree 4.21–5.00. Using this scale enabled the researcher to effectively evaluate the degree of the participants’ approval of each statement in the questionnaire, which afforded valuable insights into the participant’s perceptions, motivations and well-being. The inferential analysis in Pearson correlation coefficients was used to identify the relationships between perception, motivation, and well-being to answer question (2), and linear regression was used to determine the extent to which perception and motivation predict the well-being of students in virtual classes to answer question (3). Furthermore, the independent samples *t*-test was used to define whether the participants’ academic majors informed their perceptions, motivation, or well-being. Finally, to answer question (4), the Welch’s one-way ANOVA tests were used to determine if the participants’ age groups, experience with virtual classes and experience learning English affected their perceptions, motivations or well-being. That is, the Welch’s one-way ANOVA was used as the variances assumption of homogeneity was violated and this was measured by Levene’s test.

## Reliability

Cronbach’s Alpha and McDonald’s Omega were used to evaluate the internal consistency reliability of the study tool before analyzing the participants’ responses. This ensured the dependability and consistency of the results. All scales had high levels of internal reliability, being significantly higher than the suggested level of reliability of 0.70 for educational and psychological scales (DeVellis and Thorpe, 2022; Nunnally and Bernstein, 1994). Table 2 indicates that the Perceptions scale (1–10) with (*α* = 0.940, *ω* = 0.941) indicates an excellent level of stability for the statements used to measure the perceptions of learners regarding chat-based interaction. The Motivation scale (11–20) was also found to be very reliable (α = 0.905, ω = 0.913), supporting the consistency of the results. Equally, the well-being scale (21–30) had (α = 0.920, ω = 0.922), indicating excellent reliability for measuring the psychological experiences of well-being during virtual participation.

**Table 2:** Scale reliability statistics.

Scale	Cronbach’s *α*	McDonald’s *ω*	Interpretation
Perceptions (1–10)	0.940	0.941	Excellent
Motivation (11–20)	0.905	0.913	Excellent
Well-being (21–30)	0.920	0.922	Excellent

When combined, these findings indicate the three scales are internally consistent, and thus valuable for further analysis. The complete questionnaire items for all 30 statements are provided in the Appendix. Internal consistency alone, however, does not establish that the three scales are empirically distinct constructs; this is examined directly in the following subsection.

### Instrument validity: factor structure and discriminant validity

To assess whether perceptions, motivation, and well-being function as three empirically distinct constructs, an exploratory factor analysis (EFA) was conducted on all 30 items using minimum-residual extraction with promax rotation. Sampling adequacy was excellent (KMO = 0.923) and Bartlett’s test of sphericity was significant (χ^2^ = 3,536, df = 435, *p* < 0.001), confirming the data were suitable for factor analysis. Extraction based on eigenvalues greater than 1 yielded only two factors rather than three: the first factor absorbed almost all perception items and most motivation items, while the second factor was dominated by well-being items. When three factors were forced, the third factor was weak and poorly defined, with only two items (Q20, Q28) loading cleanly and substantial cross-loading elsewhere. A Harman’s single-factor test, conducted as a further check for common method variance, found that a single unrotated factor accounted for 52.3% of total variance, with all 30 items loading at 0.52 or above, a level conventionally taken to indicate a meaningful risk of common method bias.

To formally compare competing measurement models, confirmatory factor analyses (CFA; DWLS estimation, ordinal indicators) were run specifying one-, two-, and three-factor structures. Fit indices for all three models are reported in Table 3. Model fit improved modestly as factors were added, but none reached conventionally adequate absolute fit (RMSEA < 0.08); the three-factor model showed the best relative fit (CFI = 0.930, RMSEA = 0.101, scaled) but its latent factor correlations were very high (Perceptions–Motivation *r* = 0.940; Motivation–Well-being *r* = 0.942; Perceptions–Well-being *r* = 0.849). Applying the Fornell–Larcker criterion (comparing each factor’s average variance extracted against its correlations with the other factors) showed that discriminant validity was not established for any pair of constructs.

**Table 3:** Model fit comparison for one-, two-, and three-factor CFA models.

Model	CFI (scaled)	TLI (scaled)	RMSEA (scaled)	SRMR (robust)	χ^2^ (scaled), df
1. Factor	0.918	0.912	0.108	0.079	1,006, 405
2. Factor	0.924	0.918	0.104	0.076	962, 404
3. Factor	0.930	0.924	0.101	0.073	919, 402

All three models produced a non-positive-definite parameter covariance matrix warning, indicating borderline model identification given the very high inter-factor correlations relative to sample size (*N* = 128); this is reported as a limitation of the CFA.

Taken together, the EFA, Harman’s single-factor test, CFA model comparison, and Fornell–Larcker analysis converge on the same conclusion: perceptions, motivation, and well-being, as operationalized in this instrument, show substantial conceptual and empirical overlap and do not meet conventional standards for discriminant validity. Rather than discard the three-construct framing entirely, the researcher retains it for continuity with the study’s research questions and the wider literature, but the researcher interprets findings involving perceptions and motivation as separable predictors with appropriate caution throughout the Results and Discussion, and the researcher returns to this issue in the Limitations section.

## Results

### RQ1: what are EFL postgraduate learners’ perceptions, motivation levels, and well-being regarding chat-based interaction in synchronous virtual classrooms?

Composite scores were calculated by averaging responses across the 10 items for each construct; therefore, the standard deviations reported in Table 4 represent variability at the scale level, rather than individual item-level variability.

**Table 4:** Scale-level descriptive statistics for perception, motivation, and well-being.

Variable	*N*	Mean	SD	Level
Perceptions	128	4.15	0.881	High
Motivation	128	4.23	0.801	Very high
Well-being	128	4.17	0.836	High

As presented in Table 4, the study participants indicated a tendency for high scores across all three constructs. The mean rating for perceptions was 4.15 (SD = 0.88), indicating that most learners held positive views of chat-based interaction in synchronous virtual classes. The highest mean score was observed for motivation (M = 4.23, SD = 0.80), suggesting that chat-based features were associated with students’ self-reported willingness to participate. Well-being was also rated positively (M = 4.17, SD = 0.84), indicating that learners reported feeling emotionally comfortable, experiencing lower anxiety, and engaging positively when using chat tools. Table 5 reports the corresponding skewness, kurtosis, and observed ranges for each construct; all three show pronounced negative skew and positive kurtosis, consistent with a ceiling effect that is discussed further in the Discussion section.

**Table 5:** Skewness, kurtosis, and observed ranges for perception, motivation, and well-being (*N* = 128).

Variable	Min	Max	Skewness	Kurtosis
Perceptions	1.00	5.00	−1.44	2.37
Motivation	1.40	5.00	−1.46	2.40
Well-being	1.00	5.00	−1.35	2.11

Table 6 presents the item-level descriptive statistics for the 30 questionnaire statements, measuring EFL postgraduate learners’ perceptions, motivation, and well-being related to chat-based interaction in synchronous virtual classroom. Corresponding item-level skewness, kurtosis, and observed ranges are reported in Table 7.

**Table 6:** Item-level descriptive statistics for the chat-based interaction questionnaire (*N* = 128).

Statements	*N*	Mean	SD	Level
1.	128	4.23	1.044	Very High
2.	128	4.14	1.092	High
3.	128	4.02	1.146	High
4.	128	3.89	1.185	High
5.	128	4.02	1.112	High
6.	128	4.20	1.045	High
7.	128	4.19	1.162	High
8.	128	4.02	1.129	High
9.	128	4.45	0.937	Very High
10.	128	4.34	1.053	Very High
11.	128	4.20	1.022	High
12.	128	4.37	0.995	Very High
13.	128	4.23	1.046	Very High
14.	128	4.43	0.994	Very High
15.	128	4.16	1.041	High
16.	128	3.89	1.341	High
17.	128	4.23	1.074	Very High
18.	128	4.37	1.011	Very High
19.	128	4.33	1.013	Very High
20.	128	4.09	1.307	High
21.	128	4.26	0.958	Very High
22.	128	3.95	1.193	High
23.	128	3.91	1.207	High
24.	128	4.05	1.114	High
25.	128	4.55	0.877	Very High
26.	128	4.23	1.110	Very High
27.	128	4.17	1.102	High
28.	128	4.05	1.254	High
29.	128	3.98	1.217	High
30.	128	4.58	0.857	Very High

**Table 7:** Item-level skewness, kurtosis, and observed ranges for the chat-based interaction questionnaire (*N* = 128).

Item	Mean	SD	Min	Max	Skewness	Kurtosis
Q1	4.23	1.04	1	5	−1.35	1.10
Q2	4.14	1.09	1	5	−1.24	0.884
Q3	4.02	1.15	1	5	−1.16	0.631
Q4	3.89	1.19	1	5	−0.909	−0.061
Q5	4.02	1.11	1	5	−1.16	0.730
Q6	4.20	1.05	1	5	−1.47	1.77
Q7	4.19	1.16	1	5	−1.44	1.18
Q8	4.02	1.13	1	5	−1.16	0.739
Q9	4.45	0.937	1	5	−2.23	5.26
Q10	4.34	1.05	1	5	−1.71	2.34
Q11	4.20	1.02	1	5	−1.23	0.905
Q12	4.37	0.995	1	5	−1.82	2.95
Q13	4.23	1.05	1	5	−1.41	1.40
Q14	4.43	0.994	1	5	−2.08	4.04
Q15	4.16	1.04	1	5	−1.19	0.731
Q16	3.89	1.34	1	5	−0.852	−0.641
Q17	4.23	1.07	1	5	−1.47	1.56
Q18	4.37	1.01	1	5	−1.77	2.65
Q19	4.33	1.01	1	5	−1.72	2.51
Q20	4.09	1.31	1	5	−1.27	0.301
Q21	4.26	0.958	1	5	−1.36	1.68
Q22	3.95	1.19	1	5	−0.997	0.060
Q23	3.91	1.21	1	5	−0.856	−0.323
Q24	4.05	1.11	1	5	−1.13	0.528
Q25	4.55	0.877	1	5	−2.63	7.37
Q26	4.23	1.11	1	5	−1.51	1.57
Q27	4.17	1.10	1	5	−1.50	1.71
Q28	4.05	1.25	1	5	−1.28	0.628
Q29	3.98	1.22	1	5	−1.22	0.691
Q30	4.58	0.857	1	5	−2.50	6.56

As presented in Table 6, statements 1–10 focused on the participants’ perceptions of chat-based interactions. The highest score for the perception construct was obtained for statement 9 (“chat box made the virtual class more interactive and engaging”) with a mean value of 4.45 (SD = 0.94). This was followed by statement 10 (“Text-based interaction supported my learning when I felt hesitant to speak aloud”) with a mean value of 4.34 (SD = 1.05) and statement 1 (“Chat-box interaction helped me notice and correct my English mistakes”) with a mean value of 4.23 (SD = 1.04). The lowest score for this construct was obtained for statement 4 (“Chat-box interaction improved my English grammar”) with a mean value of 3.89 (SD = 1.19).

Statements 11–20 focused on the motivation of the participants when involved in chat-based interaction. The highest score for the motivation construct was obtained for statement 14 (“Seeing classmates participate in the chat box encourages me to join”) with a mean value of 4.43 (SD = 0.99). This was followed by statement 12 (“Texting increases my interest in participating in lesson activities”) with a mean value of 4.37 (SD = 1.00), statement 18 (“I participate more when I know chat-box participation is graded”) with a mean value of 4.37 (SD = 1.01), and statement 19 (“I participate in the chat box to improve my academic performance”) with a mean value of 4.33 (SD = 1.01). The lowest score for this construct was obtained for the statement (“I prefer texting because it reduces my shyness when participating”) with a mean value of 3.89 (SD = 1.34).

Statements 21–30 focused on the participants well-being. The highest score for the well-being construct was obtained for statement 30 (“The chat box helps protect my privacy when I am not in a quiet environment”) with a mean value of 4.58 (SD = 0.86). This was followed by statement 25 (“I feel proud when I receive positive feedback on my written answers”) with a mean value of 4.55 (SD = 0.88), statement 21 (“I feel comfortable when interacting in the chat box”) with a mean value of 4.26 (SD = 0.96), and statement 26 (“using text-based interaction makes me feel more capable of using English”) with a mean value of 4.23 (SD = 1.11). The lowest score for this construct was obtained for the statement (“I enjoy answering in the chat box more than speaking aloud”) with a mean value of 3.91 (SD = 1.21).

Taken together, the descriptive findings at both the scale level and individual item-level demonstrate a consistent tendency toward positive experiences among postgraduate EFL learners regarding chat-based interactions in synchronous virtual classroom.

### RQ2: how do EFL postgraduate learners’ perceptions of chat-based interaction in synchronous virtual classroom relate to their motivation levels and well-being?

Pearson correlation coefficients were performed to test the relationships between the three main variables, including perceptions, motivation, and psychological well-being. The results presented in Table 8 showed a statistically significant (*p* < 0.001) strong positive correlation. Table 9 presents the 95% confidence intervals for these correlations.

**Table 8:** Correlation matrix for the study variables.

Variable	Statistic	Perceptions	Motivation	Well-being
Perceptions	Pearson’s r	—		
	Df	—		
	*p*-value	—		
Motivation	Pearson’s r	0.854***	—	
	Df	126	—	
	*p*-value	<0.001	—	
Well-being	Pearson’s r	0.792***	0.867***	—
	Df	126	126	—
	*p*-value	<0.001	<0.001	—

***Correlation is significant at the 0.001. level (2-tailed).

**Table 9:** 95% confidence intervals for correlations (*N* = 128).

Correlation	*R*	95% CI lower	95% CI upper
Perceptions ↔ Motivation	0.854	0.798	0.895
Perceptions ↔ Well-being	0.792	0.717	0.849
Motivation ↔ Well-being	0.867	0.817	0.905

A very strong positive correlation was observed between perceptions and motivation (*r* = 0.854, *p* < 0.001), which reveals the more the students perceive chat-based interaction positively, the more likely they are motivated to participate. There was also a strong positive correlation between perceptions and well-being (*r* = 0.792, *p* < 0.001), which indicates that positive perceptions toward chat-based interaction provide more positive well-being experiences. A very strong positive correlation (showing the highest association) was found between motivation and well-being (*r* = 0.867, *p* < 0.001), as motivation is central to student’s well-being with chat-based interaction in virtual learning.

These findings suggest the three constructs are closely related in the learning environment of chat-based interactions online, though as noted above this may partly reflect their limited discriminant validity rather than three fully distinct processes.

### RQ3: to what extent do EFL postgraduate learners’ perceptions and motivation to engage in chat-based interaction in virtual classes predict their well-being in virtual learning environments?

The research adopted a linear regression model to determine the extent to which perceptions and motivation could be used to predict the well-being of students in virtual classes. With an R-squared (*R*^2^) score for the “well-being” component of 0.762, the model suggests explaining 76.2% of the variation in well-being variable, which is generally considered a strong fit. In other words, the total model was found to be statistically significant and accounted for 76.2% of the variability in well-being (*R*^2^ = 0.762). This *R*^2^ should be interpreted cautiously, however, given the limited discriminant validity between perceptions and motivation reported above, which means part of the shared variance may reflect overlap between highly similar predictors rather than fully independent predictive power.

Table 10 provides insights into how different predictors influence the well-being variable. It shows the strongest predictor was motivation (*β* = 0.734, *p* < 0.001), meaning that higher motivation was associated with the greatest well-being scores among learners. The predictor of perceptions also proved significant (*β* = 0.182, *p* = 0.024) where *p* < 0.05, but with a lower effect size. The result of the linear regression model indicates that motivation shows the strongest association with well-being, suggesting cognitive and emotional engagement with chat-based interaction co-occurs with higher levels of well-being; as this is a cross-sectional, single time-point design, this association cannot be interpreted as evidence of a causal or developmental process.

**Table 10:** Model coefficient for the well-being construct.

Predictor	Estimate (*β*)	SE	T	*p*
Intercept	0.310	0.196	1.58	0.117
Perceptions	0.182	0.080	2.29	0.024
Motivation	0.734	0.087	8.41	<0.001

Note on Table 10 labeling: the column originally reported as “Estimate (*β*)” contains unstandardized regression coefficients (B), not standardized *β*. The correct standardized coefficients are *β* = 0.191 (Perceptions) and *β* = 0.704 (Motivation). The 95% confidence interval for the Perceptions coefficient was [0.025, 0.339]; for Motivation, [0.562, 0.907]. Collinearity diagnostics indicated VIF = 3.69 and Tolerance = 0.271 for both predictors, below the conventional threshold of concern (VIF > 5–10), indicating moderate but not severe multicollinearity. Table 11 presents the corrected coefficients with 95% confidence intervals and collinearity statistics.

**Table 11:** Corrected coefficients with 95% confidence intervals and collinearity statistics.

Predictor	B (unstd.)	*β* (std.)	95% CI for B	VIF
Intercept	0.310	–	−0.079, 0.699	–
Perceptions	0.182	0.191	0.025, 0.339	3.69
Motivation	0.734	0.704	0.562, 0.907	3.69

Regression diagnostics were examined to assess the assumptions of linearity, normality, and homoscedasticity. The Shapiro–Wilk test indicated a significant deviation from normality (*p* < 0.001), consistent with the negative skew observed in the outcome variable. However, Q-Q plots showed only moderate deviations, and residual-versus-fitted plots revealed no clear pattern, suggesting the regression results are reasonably robust to this violation. Cook’s distance values were below 1 for all cases (M = 0.0106, max = 0.196), indicating no unduly influential cases (Field, 2024).

### RQ4: do students’ perceptions, motivation, and well-being differ in relation to demographic variables?

The demographic variables under consideration were (1) academic major, (2) age, (3) experience with virtual classes and (4) English learning experience.

To begin with, and to determine the influence of the academic major, an independent samples *t*-test was used. The independent samples *t*-test was used to define whether the students’ academic majors had any effect on their perceptions, motivation, or well-being. The results depicted in Table 12 indicated no statistically significant differences for the two academic majors (*p* > 0.05 in all variables). This implies no difference in the outcomes from chat-based interaction relative to disciplinary background.

**Table 12:** Independent samples *t*-test by academic major.

Variable	Statistic	Df	*p*
Perceptions	0.696	126	0.488
Motivation	1.078	126	0.283
Well-being	1.324	126	0.188

H_a_ μ Higher diploma in Governance and Internal Audit Assistant specialist ≠ *μ* Higher diploma in Human Resource Management.

Effect sizes (Cohen’s d) for the academic-major comparison were small for all three constructs (Perceptions *d* = 0.136; Motivation *d* = 0.210; Well-being *d* = 0.258), consistent with the non-significant t-test results reported above. Group means and standard deviations are reported in Table 13.

**Table 13:** Group means (SD) by academic major and Cohen’s *d*.

Group	Perceptions	Motivation	Well-being
Governance and internal audit (*n* = 91)	4.18 (0.802)	4.28 (0.743)	4.23 (0.786)
HR management (*n* = 37)	4.06 (1.06)	4.11 (0.930)	4.02 (0.942)
Cohen’s *d*	0.136	0.210	0.258

To test differences across age groups, a Welch’s one-way ANOVA test was used. Age had no significant effect on the perceptions or well-being, as indicated in Table 14. Nonetheless, motivation varied considerably by age group (*p* = 0.020). Games–Howell post-hoc comparisons showed this omnibus effect was attributable to a single pairwise contrast: the youngest group (23–27) reported significantly higher motivation than the oldest group (38+) (mean difference = 0.506, *p* = 0.018); no other pairwise comparisons were significant (all *p* > 0.10). This contrast did not survive Holm correction across the full family of demographic tests (p_Holm = 0.185) and should be interpreted with caution.

**Table 14:** One-way ANOVA results by age group (Welch’s).

Variable	*F*	df1	df2	*p*
Perceptions	1.71	3	58.6	0.175
Motivation	3.55	3	59.9	0.020
Well-being	2.56	3	58.7	0.063

Games–Howell post-hoc comparisons for the significant age effect on motivation identified a single significant pairwise contrast (23–27 vs. 38+, mean difference = 0.506, *p* = 0.018); all other pairwise comparisons were non-significant (*p* > 0.10 in all cases), and this contrast did not survive Holm correction across the full family of demographic tests (p_Holm = 0.185). Effect sizes for age were small across all three constructs (ω^2^ = −0.005 to 0.018). Table 15 reports the effect sizes and Holm-corrected *p*-values for the age group comparisons.

**Table 15:** Effect sizes and Holm-corrected p-values by age group.

Construct	η^2^	ω^2^	*p* (Holm)	Effect
Perceptions	0.019	−0.005	1.000	None
Motivation	0.041	0.018	0.185	Small
Well-being	0.038	0.015	0.271	Small

To assess the differences in virtual classroom experience, a Welch’s one-way ANOVA test was used. Table 16 indicated that the well-being variable showed a statistically significant effect on experience using Blackboard virtual classes (*p* = 0.016, *p* < 0.05). Post-hoc tests showed respondents who had an experience of over a year with virtual classes reported significantly greater well-being than those with less experience. This is consistent with prior exposure to digital classroom environments being associated with lower self-reported stress when interacting in a virtual chat; because the design is cross-sectional and self-reported, causal direction cannot be established. This association remained significant after Holm correction (p_Holm = 0.043), making it the most robust demographic finding in this study.

**Table 16:** One-way ANOVA results by virtual class experience (Welch’s).

Variable	*F*	df1	df2	*p*
Perceptions	0.969	2	32.7	0.390
Motivation	3.203	2	32.8	0.054
Well-being	4.684	2	35.2	0.016

Effect sizes for virtual-class experience were small-to-medium; the well-being effect remained significant after Holm correction (p_Holm = 0.043), making it the most robust demographic finding in this study. Group means (SD) are reported in Table 17.

**Table 17:** Group means (SD), effect sizes, and Holm-corrected p by virtual class experience.

Group	Perceptions	Motivation	Well-being
Less than 6 months (*n* = 55)	4.24 (0.607)	4.37 (0.613)	4.32 (0.636)
6–12 months (*n* = 13)	4.33 (0.830)	4.54 (0.823)	4.57 (0.676)
1 year+ (*n* = 60)	4.03 (1.08)	4.04 (0.909)	3.95 (0.966)
ω^2^	−0.004	0.024	0.048
p (Holm)	0.831	0.175	0.043

To test the differences between English learning experience, a Welch’s one-way ANOVA test was used. There were no important variances apparent between groups with varying English learning experience (*p* > 0.05 in all variables). Table 18 indicates that the perceptions of the participants, their level of motivation and well-being did not differ according to how long they had been learning English; whether for 3 years, 4–8 years, or more than 8 years.

**Table 18:** One-way ANOVA results by English learning experience (Welch’s).

Variable	*F*	df1	df2	*p*
Perceptions	0.564	2	74.6	0.571
Motivation	1.420	2	72.2	0.248
Well-being	1.774	2	73.4	0.177

Effect sizes for English-learning experience were small and non-significant after Holm correction for all three constructs. Group means (SD) are reported in Table 19.

**Table 19:** Group means (SD), effect sizes, and Holm-corrected p by English-learning experience.

Group	Perceptions	Motivation	Well-being
Less than 3 years (*n* = 54)	4.25 (0.907)	4.37 (0.841)	4.33 (0.882)
4–8 years (*n* = 44)	4.06 (0.889)	4.15 (0.709)	4.09 (0.760)
8 years+ (*n* = 30)	4.11 (0.832)	4.10 (0.841)	4.00 (0.833)
ω^2^	−0.007	0.008	0.014
p (Holm)	1.000	0.138	0.090

In general, the demographic information revealed no significant effects from the variables evaluated, with the exception of the virtual learning experience, which had a significant positive outcome on the Well-being.

### Sensitivity analysis and multicollinearity diagnostics

Given the researcher–instructor relationship and recruitment procedure noted in the Methods, a sensitivity analysis was conducted to assess whether straight-line or acquiescent responding influenced the main findings. Nineteen respondents (14.8%) who showed straight-line response patterns (within-person standard deviation = 0 across all 30 items, i.e., identical responses to every item) were identified and excluded, leaving *N* = 109. Correlations among the three constructs remained strong and significant in this reduced sample (Perceptions–Motivation *r* = 0.831; Motivation–Well-being *r* = 0.846; Perceptions–Well-being *r* = 0.759; all *p* < 0.001), indicating the core associational findings are not an artifact of careless responding. The regression model was also re-estimated: Motivation remained a robust, significant predictor of well-being (*B* = 0.724, *p* < 0.001), but the association between Perceptions and well-being fell just short of conventional significance once straight-line responders were removed (*B* = 0.170, *p* = 0.051, down from *p* = 0.024 in the full sample). The researcher therefore treat Motivation as the more robust predictor throughout, and note the Perceptions–well-being association should be considered fragile.

Multicollinearity between the two predictors in the regression model (Table 10) was also assessed directly via variance inflation factors. Both Perceptions and Motivation returned VIF = 3.69 (Tolerance = 0.271), which falls below the conventional threshold of concern (VIF > 5–10), indicating multicollinearity here is moderate rather than severe. Table 20 summarizes the sensitivity analysis results, comparing the full sample (*N* = 128) with the filtered sample excluding straight-line responders (*N* = 109).

**Table 20:** Sensitivity analysis: Correlations and regression coefficients with straight-line responders excluded (*N* = 109).

Estimate	Full sample (*N* = 128)	Filtered (*N* = 109)	Change
Perceptions–Motivation *r*	0.854	0.831	Held
Motivation–Well-being *r*	0.867	0.846	Held
Perceptions–Well-being *r*	0.792	0.759	Held
Perceptions → Well-being (B, *p*)	0.182, *p* = 0.024	0.170, *p* = 0.051	Lost significance
Motivation → Well-being (B, *p*)	0.734, *p* < 0.001	0.724, *p* < 0.001	Held
*R* ^2^	0.762	0.726	–

## Discussion

This paper has examined the self-reported perceptions of EFL postgraduate learners regarding chat-based interaction in synchronous virtual classroom, and the association between perceptions and the motivation and well-being of learners. In general, the results indicate a systematically positive performance for all three constructs, supporting the hypothesis that chat-based communication is associated with positive self-reported experiences in academic, motivational, and well-being domains in online EFL learning settings. The three scales used had high internal-consistency reliability; however, as noted in the Instrument Validity subsection, reliability alone does not establish that the constructs are empirically distinct or validly measured.

With regard to the first research question (“What are EFL postgraduate learners’ perceptions, motivation levels, and well-being regarding chat-based interaction in synchronous virtual classrooms?”), the descriptive findings indicated that postgraduate EFL students reported generally positive attitudes toward chat-based interaction in synchronous online classes. Respondents noted that they felt more engaged, perceived themselves as more able to use English, and reported lower hesitation when interacting using text-based communication. These self-reports align with previous studies, suggesting written online communication can be associated with reduced anxiety and increased communication among EFL learners (Knežević et al., 2025; Namaziandost et al., 2021). Respondents also reported that chat-based interactions helped them notice and correct their English errors, correlating with the output hypothesis, which proposes more learning occurs after the linguistic weaknesses in language produced are highlighted (Swain, 2005), although the current cross-sectional data cannot confirm learning gains. The responses of the female Saudi postgraduate learners suggests that the chat features may contribute to a psychologically safer learning environment for some students, in which learners can be less afraid of posting, as they do not experience the same stress as when having to communicate orally in real time. This observation is consistent with the sociocultural and interactionist viewpoints, which focus on reducing affective obstacles to enhance the opportunities for constructive interaction in language learning (Long, 1996; Vygotsky, 1978). Moreover, many students reported that text-based interaction promotes deeper thinking because learners have the time to process received information and compose additional output (Chun et al., 2016). This reflective aspect of chat-based interaction may explain the positive perception ratings in the current study.

In answer to the second research question (“How do EFL postgraduate learners’ perceptions of chat-based interaction in synchronous virtual classroom relate to their motivation and well-being?”), the results indicated that all three constructs were strongly and significantly related, with motivation exhibiting the highest relationship with well-being. However, as detailed in the Instrument Validity subsection, these high inter-construct correlations also reflect limited discriminant validity between perceptions and motivation, and should be interpreted with this in mind. These findings suggest that in situations when learners feel that chat-based interaction is supportive and involving, they are more likely to report positive motivational and emotional experiences, though intrinsic motivation itself was not directly operationalized as a distinct Self-Determination Theory construct in this questionnaire. These correspond to Self-Determination Theory, which holds that autonomy, competence, and relatedness are major motivators and positively affect well-being (Ryan and Deci, 2000). Participation in the form of chat appears to encourage greater autonomy, by giving learners the opportunity to answer at their own pace. Chat-based participation may plausibly support learners’ sense of competence through successful communication attempts, and relatedness through opportunities for socialization in the virtual classroom, although competence and relatedness were not directly measured in this study.

In answering the third research question (“To what extent do EFL postgraduate learners’ perceptions and motivation to engage in chat-based interaction in virtual classes predict their well-being in virtual learning environments?”), the linear regression model analysis provided additional detail, finding motivation to be the strongest predictor of well-being. This indicates that well-being in online learning situations is not merely a result of technological properties, but is associated with the motivational experiences that those properties facilitate. In cases where chat-based tools alleviate communication anxiety and facilitate meaningful peer interaction, learner motivation is positively associated and self-reported well-being outcomes are more positive. However, as this is a cross-sectional design, these patterns reflect association rather than a demonstrated causal sequence. These results coincide with the findings of recent studies that focused on the key role of motivation to maintain engagement and emotional preparedness in the virtual learning setting (Jiang et al., 2024; Xu and Rahim, 2025). Also, the importance of well-being as an outcome of online learning is emphasized in previous research on multimodal interaction tools in online education; specifically chat, as it contributes to the development of the sense of social presence and community among learners (Hampel, 2019). This further supports the findings of the current study.

In relation to the fourth research question (“Do students’ perceptions, motivation, and well-being differ in relation to demographic variables?”), the results did not show any significant differences arising from academic major or English-learning experience. Age showed a significant omnibus effect on motivation (*p* = 0.020), though this was driven by a single pairwise contrast between the youngest (23–27) and oldest (38+) groups that did not survive Holm correction for multiple comparisons (p_Holm = 0.185); no significant effects were found for perceptions or well-being. This suggests that learners’ self-reported experiences with chat-based interaction were broadly similar across most of the demographic groups examined, rather than indicating that chat-based interaction is beneficial to all learners irrespective of background. Nonetheless, it emerged that the participants’ experiences in virtual classes were associated with their well-being. The learners with more prior experience of online learning environments, reported experiencing greater emotional comfort and well-being. This can be explained by a decreased cognitive load, greater acquaintance with online resources, and the emotional regulation over time. These results align with previous studies, which have shown technological confidence and frequent exposure have a positive effect on online interactions and learners’ comfort (Means et al., 2014). The insignificant distinctions between the age and acquisition of English learning experiences are indicative of the fact that the chat-based interaction offers an equitable learning platform, where students from varied backgrounds can comfortably participate in interactions.

### Theoretical implications for CALL research, pedagogy, and policy

Theoretically, the findings point to the value of conceptualizing chat-based interactions, not merely as a technological feature, but as a pedagogically meaningful mode of communication that is associated with aspects social presence, affective engagement, and communicative competence. The close association between motivation and well-being suggests that affective variables deserve attention when developing online language learning models, because of the growing recognition of the role of positive psychology in second language acquisition studies (MacIntyre et al., 2019; Seligman, 2011; Seligman and Csikszentmihalyi, 2000).

The pedagogical implications of the findings suggest that educators may consider including chat-based interactions in regular EFL teaching courses. Chat tools may foster the involvement of less talkative students, encourage collaborative meaning-making, and provide low-pressure language production. In situations where cultural issues might affect classroom interactions, as in the case of Saudi Arabian postgraduate women, chat-based communication offers a platform for inclusive and effective communication.

At the policy and curriculum level, the results imply that institutions could consider including structured chat activities when designing virtual courses, instead of relying on the discretion of the instructors. Curriculum designers and policymakers may consider promoting digital literacy training for teachers and learners to enable them to use the synchronous tools effectively. Looking at the inclusion of multimodal interaction activities, including guided chat discussion, collaborative text-based problem-solving, and reflective chat prompts, could plausibly improve learner engagement, social presence, and well-being as suggested by the positive association of perceptions with motivation and well-being.

## Conclusion

This research aimed to examine the perceptions, motivation, and well-being of EFL postgraduate learners in connection with chat-based interactions during synchronous virtual classes in Saudi higher education. The results showed the students had high positive perceptions of chat-based interaction, as they reported high motivation and positive well-being. There was also a meaningful relationship between the three constructs, whereby motivation was identified as the greatest predictor of well-being. These findings provide exploratory evidence for the pedagogical significance of chat-based interaction and suggest that written synchronous interaction is associated with decreased anxiety and enhanced engagement, and enrich emotional experiences in online EFL communication classes.

The research adds to existing literature in this area in a number of significant ways. It explains chat-based interaction is a psychologically and pedagogically significant mode of communication. Methodologically, it provides quantitative data on a sample of female Saudi postgraduates, a group largely underrepresented in previous research. Practically, the results emphasize the importance of instructors including chat-based activities to create supportive, engaging, and emotionally safe learning environments in virtual classrooms.

Despite its value, this study was subject to several limitations. It was conducted in the context of a single Saudi university with only female postgraduates, which limits generalizability; the two academic programs sampled were also unevenly represented (28.9% vs. 71.1%). The response rate was 64.6% (128 of 198 invited students), and the possibility of nonresponse bias cannot be ruled out, as non-respondents may differ systematically from respondents in ways this study cannot capture. Also, the study relied on self-reported data, which can be influenced by perceived social desirability or recall bias. A sensitivity analysis excluding 19 respondents (14.8%) who showed straight-line response patterns was conducted; the core correlational findings held, but the association between perceptions and well-being lost statistical significance (*p* = 0.051), indicating some findings are sensitive to careless responding. Additionally, exploratory and confirmatory factor analyses indicated that perceptions, motivation, and well-being did not show adequate discriminant validity (latent factor correlations of 0.85–0.94; Fornell–Larcker criterion not met for any pair), and a Harman’s single-factor test found one general factor accounted for 52.3% of total variance. These results suggest the three constructs, as operationalized here, substantially overlap and may partly reflect a broader general orientation toward chat-based interaction rather than three fully separable constructs; findings involving perceptions and motivation as distinct predictors should therefore be interpreted with caution. Relatedly, the well-being items in this instrument were designed to capture affective comfort and preference for chat-based interaction specifically, and do not fully correspond to the broader conceptualizations of psychological well-being (e.g., PERMA, flourishing) cited in the Introduction; for instance, item 30 (“The chat box helps protect my privacy when I am not in a quiet environment”) reflects situational comfort and practical convenience rather than psychological well-being as conventionally defined, illustrating this conceptual gap. Future studies should consider validated instruments such as the WHO-5, the PERMA-Profiler, or the L2 well-being scale of Zhou and Liu (2025) to measure this construct more comprehensively. Similar research in the future should be conducted using mixed methods, such as classroom observations or interviews, to gather more insights into the interactional behaviors of the learners. Researchers could also employ longitudinal designs to capture changes in motivation and well-being as learners become more familiar with virtual platforms, or comparative studies identifying variance between text-based, audio-based, and video-based interactions.

Additionally, this study focused specifically on learners’ perceptions of chat-based interaction. However, the positive ratings may partly reflect overall satisfaction with the Blackboard course, the instructor, or the online learning experience rather than chat interaction exclusively. The study did not collect data on the frequency or quality of chat use, nor did it compare chat-based interaction with other modes of participation. Future research should isolate chat-specific effects through experimental designs or chat log analysis.

In summary, the results present exploratory evidence to indicate that chat-based interaction is associated with positive motivational and emotional components of online learning in EFL. By identifying its potential, it is hoped the study will encourage teachers and schools to integrate it to deliver more active, self-confident, and psychologically healthy learning online.

## Data Availability

The original contributions presented in the study are included in the article/Supplementary material, further inquiries can be directed to the corresponding author.
