# Supplementary material for: Chat-based interaction in virtual classes: EFL students’ perceptions, motivation, and well-being
Source: Front Psychol. 2026 Aug 25;17:1807648. doi: 10.3389/fpsyg.2026.1807648 (PMC13549909; doi:10.3389/fpsyg.2026.1807648)
Supplement: Supplementary file 1 [file Supplementary_file_1.docx]

Appendix

Item-level descriptive statistics in detail for chat-based interaction questionnaire (N = 128)

| Perception (Statements 1–10) | | | | | | | | | |
| --- | --- | --- | --- | --- | --- | --- | --- | --- | --- |
| Statement | **N** | **Strongly Disagree**  **n (%)** | **Disagree**  **n (%)** | **Neutral**  **n (%)** | **Agree**  **n (%)** | **Strongly Agree**  **n (%)** | **Mean** | **SD** | **Level** |
| 1. Chat-box interaction helped me notice and correct my English mistakes. | 128 | 3  (2.3%) | 9  (7.0%) | 13 (10.2%) | 34 (26.6%) | 69 (53.9%) | 4.23 | 1.04 | Very High |
| 2. Reading classmates’ chat messages helped me learn new language forms. | 128 | 5  (3.9%) | 6  (4.7%) | 20 (15.6%) | 32 (25.0%) | 65 (50.8%) | 4.14 | 1.09 | High |
| 3. Chat-box interaction improved my English spelling. | 128 | 7  (5.5%) | 7  (5.5%) | 19 (14.8%) | 38 (29.7%) | 57 (44.5%) | 4.02 | 1.15 | High |
| 4. Chat-box interaction improved my English grammar. | 128 | 7  (5.5%) | 11  (8.6%) | 22 (17.2%) | 37 (28.9%) | 51 (39.8%) | 3.89 | 1.19 | High |
| 5. Chat-box interaction increased my English vocabulary. | 128 | 6  (4.7%) | 8  (6.2%) | 17 (13.3%) | 43 (33.6%) | 54 (42.2%) | 4.02 | 1.11 | High |
| 6. Answering in the chat box after listening to the audio improved my listening comprehension. | 128 | 5  (3.9%) | 5  (3.9%) | 14 (10.9%) | 39 (30.5%) | 65 (50.8%) | 4.20 | 1.05 | High |
| 7. The chat box provided more opportunities to practice English than verbal participation. | 128 | 7  (5.5%) | 7  (5.5%) | 13 (10.2%) | 29 (22.7%) | 72 (56.2%) | 4.19 | 1.16 | High |
| 8. Asking for clarification questions in the chat box improved my understanding of lessons. | 128 | 7  (5.5%) | 6  (4.7%) | 20 (15.6%) | 40 (31.2%) | 55 (43.0%) | 4.02 | 1.13 | High |
| 9. The chat box made the virtual class more interactive and engaging. | 128 | 5  (3.9%) | 1  (0.8%) | 7  (5.5%) | 34 (26.6%) | 81 (63.3%) | 4.45 | 0.94 | Very High |
| 10. Text-based interaction supported my learning when I felt hesitant to speak aloud. | 128 | 5  (3.9%) | 4  (3.1%) | 14 (10.9%) | 24 (18.8%) | 81 (63.3%) | 4.34 | 1.05 | Very High |
| Motivation (Statements 11–20) | | | | | | | | | |
| Statement | **N** | **Strongly Disagree**  **n (%)** | **Disagree**  **n (%)** | **Neutral**  **n (%)** | **Agree**  **n (%)** | **Strongly Agree**  **n (%)** | **Mean** | **SD** | **Level** |
| 11. Texting in the chat box makes learning English more enjoyable for me. | 128 | 3  (2.3%) | 6  (4.7%) | 20 (15.6%) | 32 (25.0%) | 67 (52.3%) | 4.20 | 1.02 | High |
| 12. Texting increases my interest in participating in lesson activities. | 128 | 4  (3.1%) | 5  (3.9%) | 9 (7.0%) | 32 (25.0%) | 78 (60.9%) | 4.37 | 1.00 | Very High |
| 13. I feel motivated to participate by text even when it is not required. | 128 | 4  (3.1%) | 6  (4.7%) | 16 (12.5%) | 32 (25.0%) | 70 (54.7%) | 4.23 | 1.05 | Very High |
| 14. Seeing classmates participate in the chat box encourages me to join. | 128 | 5  (3.9%) | 3  (2.3%) | 8 (6.2%) | 28 (21.9%) | 84 (65.6%) | 4.43 | 0.99 | Very High |
| 15. Expressing myself through writing increases my motivation to learn English. | 128 | 3  (2.3%) | 8  (6.2%) | 18 (14.1%) | 35 (27.3%) | 64 (50.0%) | 4.16 | 1.04 | High |
| 16. I prefer texting because it reduces my shyness when participating. | 128 | 9  (7.0%) | 17 (13.3%) | 17 (13.3%) | 21 (16.4%) | 64 (50.0%) | 3.89 | 1.34 | High |
| 17. I find texting easier because it avoids technical issues with microphones. | 128 | 5  (3.9%) | 6  (4.7%) | 14 (10.9%) | 33 (25.8%) | 70 (54.7%) | 4.23 | 1.07 | Very High |
| 18. I participate more when I know chat-box participation is graded. | 128 | 4  (3.1%) | 5  (3.9%) | 11  (8.6%) | 28 (21.9%) | 80 (62.5%) | 4.37 | 1.01 | Very High |
| 19. I participate in the chat box to improve my academic performance. | 128 | 4  (3.1%) | 6  (4.7%) | 9  (7.0%) | 34 (26.6%) | 75 (58.6%) | 4.33 | 1.01 | Very High |
| 20. Using emojis or reactions makes me more willing to participate. | 128 | 10 (7.8%) | 11  (8.6%) | 10  (7.8%) | 23 (18.0%) | 74 (57.8%) | 4.09 | 1.31 | High |
| Well-being (Statements 21–30) | | | | | | | | | |
| Statement | **N** | **Strongly Disagree**  **n (%)** | **Disagree**  **n (%)** | **Neutral**  **n (%)** | **Agree**  **n (%)** | **Strongly Agree**  **n (%)** | **Mean** | **SD** | **Level** |
| 21. I feel comfortable when interacting in the chat box. | 128 | 3 (2.3%) | 3  (2.3%) | 19 (14.8%) | 36 (28.1%) | 67 (52.3%) | 4.26 | 0.96 | Very High |
| 22. Texting in the chat box reduces my anxiety about participating. | 128 | 7 (5.5%) | 11  (8.6%) | 19 (14.8%) | 36 (28.1%) | 55 (43.0%) | 3.95 | 1.19 | High |
| 23. I enjoy answering in the chat box more than speaking aloud. | 128 | 6 (4.7%) | 14 (10.9%) | 21 (16.4%) | 32 (25.0%) | 55 (43.0%) | 3.91 | 1.21 | High |
| 24. I feel more confident about my answers when I type them. | 128 | 5 (3.9%) | 10  (7.8%) | 16 (12.5%) | 40 (31.2%) | 57 (44.5%) | 4.05 | 1.11 | High |
| 25. I feel proud when I receive positive feedback on my written answers. | 128 | 4 (3.1%) | 2  (1.6%) | 3  (2.3%) | 29 (22.7%) | 90 (70.3%) | 4.55 | 0.88 | Very High |
| 26. Using text-based interaction makes me feel more capable of using English. | 128 | 6 (4.7%) | 6  (4.7%) | 13 (10.2%) | 31 (24.2%) | 72 (56.2%) | 4.23 | 1.11 | Very High |
| 27. I feel connected to my classmates when communicating through the chat box. | 128 | 7 (5.5%) | 4  (3.1%) | 14 (10.9%) | 38 (29.7%) | 65 (50.8%) | 4.17 | 1.10 | High |
| 28. Emojis and reactions help me express my feelings comfortably in class. | 128 | 11 (8.6%) | 5  (3.9%) | 16 (12.5%) | 31 (24.2%) | 65 (50.8%) | 4.05 | 1.25 | High |
| 29. Texting helps me avoid embarrassment and participate more freely. | 128 | 11 (8.6%) | 3  (2.3%) | 20 (15.6%) | 37 (28.9%) | 57 (44.5%) | 3.98 | 1.22 | High |
| 30. The chat box helps protect my privacy when I am not in a quiet environment. | 128 | 3 (2.3%) | 2  (1.6%) | 7  (5.5%) | 22 (17.2%) | 94 (73.4%) | 4.58 | 0.86 | Very High |
